# Identification of Potential Biomarkers for Rheumatoid Arthritis Based on Integrated Bioinformatics and Single-Cell RNA-Seq

**DOI:** 10.3390/genes17070828

**Published:** 2026-07-21

**Authors:** Jinling Zhang, Ke Han

**Affiliations:** 1 School of Pharmacy, Harbin University of Commerce, Harbin 150076, China; 2 Heilongjiang Provincial Key Laboratory of Fluid Engineering Equipment and Digital Intelligence Technology, Harbin University of Commerce, Harbin 150029, China; 3 School of Computer and Information Engineering, Harbin University of Commerce, Harbin 150028, China

**Keywords:** rheumatoid arthritis, mendelian randomization, single-cell RNA sequencing, druggable genes, machine learning

## Abstract

Background/objectives: Rheumatoid arthritis (RA) is a chronic autoimmune disease that causes progressive joint damage and systemic complications. Despite multiple treatment options, many patients fail to achieve sustained remission. Our study aimed to integrate bioinformatics and single-cell RNA-seq analyses to identify potential biomarkers and therapeutic targets and explore bioactive compounds from traditional Chinese medicine (TCM). Methods: We integrated gene expression quantitative trait loci (eQTL), protein quantitative trait loci (pQTL), and genome-wide association study (GWAS) data for RA using two-sample Mendelian randomization to identify causal druggable genes. Bulk transcriptomics and machine learning were used for candidate gene screening and validation, while single-cell RNA-seq analysis characterized cell type-specific expression and functional relevance. TCM compound screening, molecular docking, and molecular dynamics (MD) simulations were subsequently performed. Results: *CXCL6*, *IFNG*, and *SLAMF1* were identified as RA-associated candidate targets with distinct cell type-specific expression patterns, strong immune associations, and favorable diagnostic performance. Functional analyses linked these genes to immune activation and intercellular communication. In silico analyses prioritized sesamin, (+)-Ganoderic acid Mf, and (24R)-saringosterol as candidate compounds, with the IFNG–(+)-Ganoderic acid Mf complex showing stable behavior during MD simulation. Conclusions: This integrative framework identified *CXCL6*, *IFNG*, and *SLAMF1* as candidate biomarkers and druggable targets for RA. Sesamin, (+)-Ganoderic acid Mf, and (24R)-saringosterol warrant further experimental evaluation. These findings provide a basis for future mechanistic and translational studies.

## 1. Introduction

Rheumatoid arthritis (RA) is a chronic autoimmune disorder and affects about 1% of the world’s population [1,2]. The chronic synovitis of the small joints is the main pathological feature of this disorder, which further leads to pain and swelling and is associated with a gradual loss of joint function. As the disease progresses, it can lead to deformity and disability of the joints, and premature death, imposing an economic and social burden on society [3,4]. Although there have been significant advances in targeted therapies, RA is incurable and still needs to be treated for a long time with medications known as disease-modifying antirheumatic drugs (DMARDs), corticosteroids, nonsteroidal anti-inflammatory drugs (NSAIDs) and biologics. Given the significant proportion of patients who are non-responsive and do not achieve disease remission, it is important to identify novel therapeutic targets and candidate biomarkers to support personalized management of the disease.

To develop effective therapeutic strategies, it is necessary to accurately identify therapeutic targets and understand their roles in disease pathogenesis. However, the traditional procedure for drug development is complex and expensive. Combining genomic data has significantly improved target identification [5], and gene-based therapies have a better clinical success rate than conventional therapies [6,7,8]. Some genes are considered druggable since their proteins may be modified by small molecules or biologics [9,10]. Genome-wide association studies (GWAS) can be used in conjunction with molecular quantitative trait loci (molQTLs, e.g., expression quantitative trait loci (eQTLs) and protein quantitative trait loci (pQTLs)), allowing genetic variants associated with disease susceptibility to be pinpointed [11,12]. Mendelian randomization (MR) can infer potential causal relationships between exposures and disease outcomes, reducing confounding bias and reliance on conventional observational clinical data by using genetic variants as instrumental variables [13,14]. By combining GWAS and MR analyses with target discovery, a solid framework is established to uncover candidate treatment targets for RA.

Most existing studies rely on gene expression data from bulk tissues, which may mask cell-type-specific gene expression patterns and the functional contributions of different cell populations to RA pathogenesis. The advent of single-cell RNA sequencing (scRNA-seq) enables quantification of gene expression across distinct cell populations, revealing distinct cellular contributions to RA pathogenesis [15,16,17,18]. In RA, follicular helper T (Tfh) cells promote B-cell activation and differentiation into plasma cells, and dysregulated Tfh-cell responses may facilitate autoantibody production. Moreover, circulating Tfh (cTfh) cells display substantial transcriptional heterogeneity and comprise multiple functional subsets with diverse immunological properties. Therefore, scRNA-seq analysis of peripheral blood Tfh cells provides a biologically relevant context for evaluating the expression patterns and potential functional roles of core druggable genes. Yet, research that integrates GWAS, MR, bulk transcriptomics, and Tfh-cell-specific scRNA-seq to prioritize potential therapeutic targets in RA remains scarce.

Previous studies have linked C-X-C motif chemokine ligand 6 (*CXCL6*), interferon gamma (*IFNG*), and signaling lymphocytic activation molecule family member 1 (*SLAMF1*) to immune dysregulation in RA. *CXCL6* contributes to neutrophil recruitment and synovial inflammation, *IFNG* is involved in T-cell-mediated inflammatory responses and immune-cell interactions, and *SLAMF1* participates in immune-cell activation and intercellular communication. However, their causal associations with RA risk, diagnostic relevance, and cell type-specific expression patterns have not been systematically characterized within an integrated genetic and transcriptomic framework.

**Figure 1:**
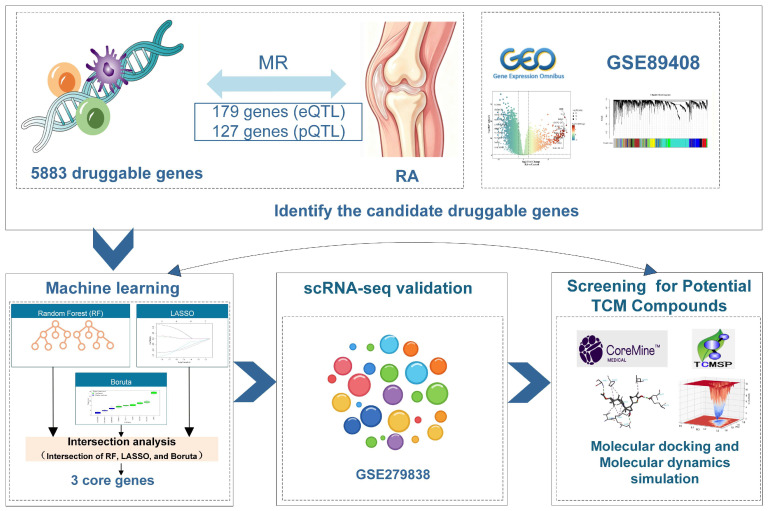
Flowchart of the study design.

To fill this research gap, we hypothesized that integrating genetic causal evidence with bulk and single-cell transcriptomic profiles would enable the identification of robust druggable genes associated with RA and clarify their disease-relevant cellular signatures within the Tfh-cell compartment. Accordingly, this study aimed to identify RA-associated druggable genes, evaluate their diagnostic value, and characterize their single-cell transcriptional patterns and potential functional relevance. We further explored traditional Chinese medicine (TCM)-derived compounds predicted to interact with these targets. An overview of the study design is presented in Figure 1.

## 2. Materials and Methods

### 2.1. Data Sources and Mendelian Randomization Analysis

We retrieved human transcriptome data from the Gene Expression Omnibus (GEO) database using the keyword “rheumatoid arthritis”. Two synovial tissue datasets were used: GSE89408 (150 RA patients, 23 healthy controls [HC], training set) and GSE55235 (10 RA patients, 10 HC, validation set). Probe IDs were mapped to gene symbols, and the mean expression value was used for genes with multiple probes. We additionally incorporated single-cell transcriptomic profiles (GSE279838) of peripheral blood Tfh cells from 3 RA patients and 3 HC donors. Detailed information on all datasets used in this study is provided in Table S1.

We obtained 5883 druggable gene records from the study by Su et al. [19]. *Cis*-eQTL data were sourced from the online eQTLGen Consortium (https://www.eqtlgen.org; accessed on 5 June 2025; *n* = 31,684), and cis-pQTL data were obtained from the deCODE project (https://www.decode.com/summarydata; accessed on 5 June 2025, *n* = 35,559, 4719 plasma proteins). Variants were filtered by *p <* 1 × 10^−5^, F > 10, and minor allele frequency > 0.01, with linkage disequilibrium (LD) pruning (*r*^2^ < 0.001, 100 kb window) using European LD references from the ieugwasr package (version 1.0.1). GWAS summary statistics for RA (ebi-a-GCST90018910; 8255 cases, 409,001 controls) were obtained from the Integrative Epidemiology Unit (IEU) database (*p* < 5 × 10^−8^).

Two-sample MR was performed using the TwoSampleMR package (version 0.6.6) [20] to assess the causal associations of druggable genes with RA risk. The inverse-variance weighted (IVW) method was applied to estimate causal effect sizes, with findings expressed as odds ratios (ORs) with 95% confidence intervals. Heterogeneity was tested using Cochran’s Q statistic (*p* < 0.05), while MR-Egger regression and Mendelian Randomization Pleiotropy RESidual Sum and Outlier were applied to detect horizontal pleiotropy to examine the causal relationship between the selected instrumental variables and outcome data.

### 2.2. Differential Expression and Co-Expression Network Analysis

Differentially expressed genes (DEGs) were identified using the DESeq2 package (version 1.46.0) [21] by comparing RA with HC samples, with thresholds of *p* < 0.05 and |log_2_FC| > 1. Volcano plots and heatmaps of the top 10 dysregulated genes were created using ggplot2 and pheatmap. We carried out weighted gene co-expression network analysis (WGCNA) on the 5000 genes showing the highest variability (ranked by median absolute deviation), aiming to uncover co-expressed gene modules linked to RA. To meet the scale-free topology criterion (*R*^2^ > 0.8), the optimal soft-thresholding power was determined using the pickSoftThreshold function. An adjacency matrix was converted to a Topological Overlap Matrix (TOM) for hierarchical clustering and dynamic tree cutting. We examined the correlations of module eigengenes with clinical characteristics, and modules with an absolute correlation > 0.5 and *p <* 0.05 were considered as key RA-related modules.

### 2.3. Identification, Functional Enrichment, and PPI Network Construction

Causal druggable genes identified from eQTL and pQTL analyses (OR > 1 or OR < 1) were intersected with DEGs and WGCNA modules to obtain upregulated and downregulated key gene sets. We conducted functional enrichment analysis using the clusterProfiler package (version 4.2.2) [22], covering three Gene Ontology (GO) categories (biological process, cellular component, and molecular function) and Kyoto Encyclopedia of Genes and Genomes (KEGG) pathway terms. Additionally, protein–protein interaction (PPI) network analysis was conducted on the overlapping gene set using the STRING (Search Tool for the Retrieval of Interacting Genes/Proteins) database (https://string-db.org/, accessed on 5 June 2025), with a minimum confidence score > 0.15 for protein interactions. Highly connected subnetworks and potential hub genes were screened with the Cytoscape MCODE plugin (version 3.10.0).

### 2.4. Machine Learning Identification of Key Druggable Genes

Three machine learning approaches, namely least absolute shrinkage and selection operator (LASSO), random forest (RF), and Boruta, were used to screen key druggable genes. LASSO was performed using the glmnet package (version 4.1-7, binomial family, 10-fold cross-validation). The RF algorithm (randomForest package, version 4.7-1.1) ranked genes by MeanDecreaseAccuracy using 70 trees. The Boruta package (version, 8.0.0) was then used to select consistently important features across iterations. The overlapping genes jointly identified by the three algorithms were defined as core druggable genes.

### 2.5. Validation and Functional Assessment of Key Druggable Genes

The expression levels of key druggable genes were validated in RA and HC via the Wilcoxon rank-sum test in both the training set (GSE89408) and validation set (GSE55235). For differentially expressed genes that show consistent changing trends across datasets, box plots are generated for visualization using ggplot2. Receiver operating characteristic (ROC) analysis was used to evaluate diagnostic performance, and genes with area under the ROC curve (AUC) > 0.7 were retained. GGally was used to analyze the correlations among genes. For further functional insight, we conducted gene set enrichment analysis (GSEA) based on KEGG pathway sets retrieved from Molecular Signatures Database. RA samples were then stratified into high-expression and low-expression groups according to the median expression level of each core gene. We plotted the top three significantly upregulated and downregulated biological pathways (*p* < 0.05). Finally, a nomogram was established using the rms package (version 6.8-0) to evaluate the predictive efficiency of key genes for RA risk. The predictive performance of the model was evaluated via calibration curves, Decision Curve Analysis (DCA), Clinical Impact Curves (CICs) and ROC analysis.

### 2.6. Immune Cell Infiltration and Correlation with Key Druggable Genes

Immune cell infiltration in RA was evaluated using single-sample gene set enrichment analysis (ssGSEA) based on predefined immune cell marker gene sets. To conduct between-group comparisons, the Wilcoxon rank-sum test was applied (*p* < 0.05). Pearson correlation assessed associations among immune cells and between core druggable gene expression and immune infiltration.

### 2.7. Single-Cell RNA-Seq Data Processing and Cell Subpopulation Annotation

The Seurat package (version 5.1.0) [23] was applied with standard parameters (min.cells = 3, min.features = 200) to process single-cell transcriptomic data from three RA samples and three HC samples (GSE279838). We only selected cells with mitochondrial content below 10% and the number of detected genes ranging from 300 to 4000 for subsequent analyses. Following normalization of raw transcript counts, the top 2000 highly variable genes were selected. Next, we conducted principal component analysis (PCA) and selected the most biologically meaningful principal components for subsequent analyses. We adjusted for batch effects via the Harmony package (version 1.2.0) [24].

We performed cellular clustering utilizing the FindNeighbors, FindClusters, RunUMAP, and RunTSNE functions with a resolution parameter of 0.5. Cell subpopulations were annotated according to marker genes from the Annotation of Cell Types database [25]. Scores of druggable genes were computed via the AddModuleScore function, and the differences between RA and control groups were subsequently evaluated. Cells with the largest score differences were further classified according to the differences in gene expression.

Additionally, we employed the Monocle package (version 2.30.1) [26] for pseudotime analysis to infer cell differentiation trajectories and identify genes related to cell transition processes. Receptor-ligand interactions and intercellular communication networks were inferred and visualized using CellChat [27].

### 2.8. Prediction of Traditional Chinese Medicine

As an exploratory complement to our target discovery efforts, we used public databases containing curated target–herb–compound associations to screen for TCM compounds relevant to the core druggable genes. The COREMINE database [28] was used to screen relevant TCMs (*p* < 0.05), and their active compounds were obtained from the Traditional Chinese Medicine Systems Pharmacology Database and Analysis Platform (TCMSP) database [29] based on the criteria of oral bioavailability (OB ≥ 30%) and drug-likeness (DL ≥ 0.18). PubChem [30] provided information on chemical structures, while the ADMET database [31] was used to predict drug properties, including absorption, distribution, metabolism, excretion, and toxicity.

### 2.9. Molecular Docking and Molecular Dynamics Simulation

The three-dimensional spatial conformations of core druggable proteins serving as drug development targets were obtained via the Protein Data Bank (PDB) [32]. In addition, we used the CB-DOCK2 platform [33] to perform molecular docking between proteins and screened active compounds, and to evaluate their interaction strength by calculating binding free energy.

To gain deeper insight into how firmly the two components associate, a molecular dynamics (MD) simulation lasting 100 ns was performed on the IFNG–(+)-Ganoderic acid Mf assembly using the GROMACS software package (version 2024.5). The protein was modeled with the CHARMM36 force field, while the ligand was treated with General AMBER Force Field 2. Prior to the main simulation, the protein-ligand complex was immersed in a water box parameterized by the three-point transferable intermolecular potential model and then neutralized with ions (2 fs timestep). The particle mesh Ewald method was used to treat distant electrostatics. Throughout the simulation period, the assessment of structural stability and binding properties relied on multiple computational parameters: root mean square deviation (RMSD), root mean square fluctuation (RMSF), radius of gyration (Rg), solvent-accessible surface area (SASA), and binding free energy.

### 2.10. Statistical Analysis

All quantitative computations ran in R (version 4.1.2). Normality was not formally assessed, and differences between the two groups, including gene expression levels, immune infiltration scores, and other quantitative characteristics, were evaluated using the Wilcoxon rank-sum test, a non-parametric method that does not require normally distributed data. Statistical significance in graphical representations was denoted as follows: ns (*p* > 0.05), * (*p* < 0.05), ** (*p* < 0.01), *** (*p* < 0.001), and **** (*p* < 0.0001).

## 3. Results

### 3.1. Integrated Identification of Candidate Druggable Genes in RA

**Figure 2:**
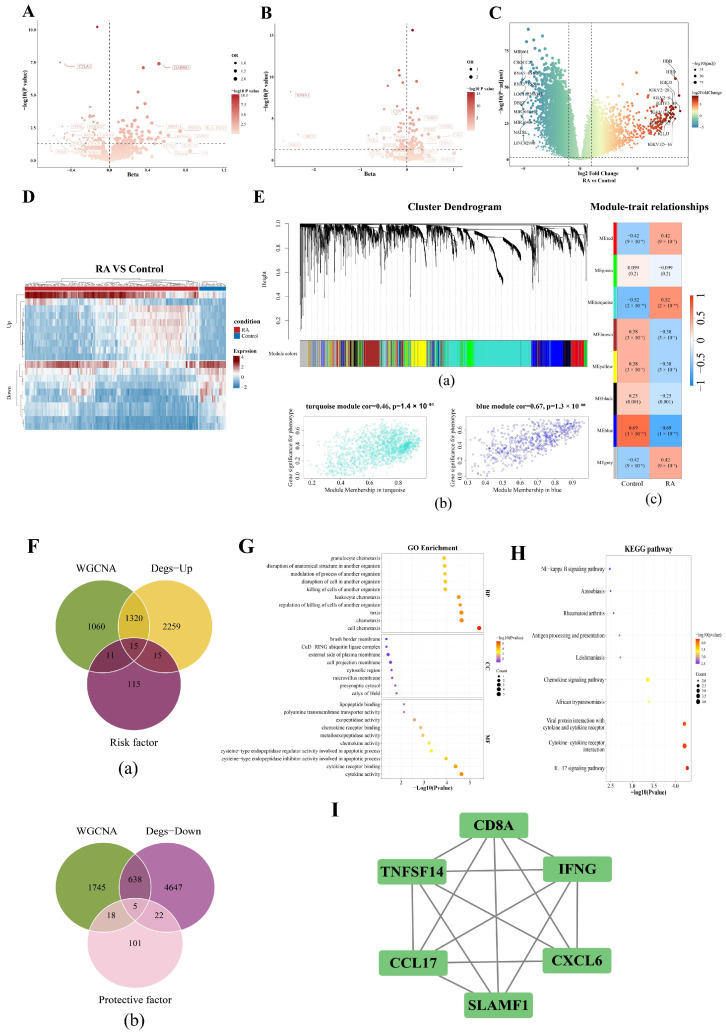
Identification of potential RA-associated genes. (**A**) Volcano plot of eQTL MR analysis. (**B**) Volcano plot of pQTL MR analysis. (**C**) Volcano plot of differentially expressed genes. (**D**) Heatmap showing the top 10 significantly upregulated and downregulated genes. (**E**) (**a**–**c**) Hierarchical clustering dendrogram (Dynamic Tree Cutting), scatter plots of gene significance versus module membership, and heatmap of module-trait relationships. (**F**) (**a**,**b**) Intersection analysis identified 15 risk genes from 156 risk genes, 3609 upregulated DEGs, and 2406 WGCNA module genes, as well as 5 protective genes from 146 protective genes, 5312 downregulated DEGs, and 2406 WGCNA module genes. (**G**) Bubble plot of GO enrichment analysis for the 15 risk-associated genes. (**H**) Bubble plot of KEGG enrichment analysis for the 15 risk-associated genes. (**I**) PPI network of the six candidate druggable genes.

Based on *cis*-eQTL and *cis*-pQTL data, 179 and 127 druggable genes were significantly associated with RA, respectively, with 4 overlapping genes removed to avoid redundancy, resulting in a total of 302 unique genes. Two-sample MR analysis identified causal relationships between these genes and RA, with OR < 1 indicating protective effects and OR > 1 indicating risk. Sensitivity analyses confirmed result robustness without significant heterogeneity or pleiotropy (Figure 2A,B). Differential expression analysis of the GSE89408 dataset revealed 8921 DEGs (3609 upregulated, 5312 downregulated; Figure 2C,D). WGCNA identified two key gene modules (turquoise and blue) strongly correlated with RA, encompassing 2406 genes (Figure 2E and Figure S1A–C). Intersection of MR-derived causal genes, DEGs, and WGCNA modules yielded 20 candidate druggable genes,15 risk and 5 protective (Figure 2F). GO and KEGG enrichment analyses showed that risk genes were mainly involved in immune and inflammatory pathways (e.g., nuclear factor kappa B (NF-κB), interleukin-17 (IL-17), chemokine signaling), whereas protective genes were enriched in metabolic and mineral absorption pathways related to immune regulation and bone protection (Figure 2G,H and Figure S2A,B). PPI network analysis further highlighted six hub genes (*CD8A*, *CCL17*, *TNFSF14*, *CXCL6*, *IFNG*, and *SLAMF1*) as potential therapeutic targets (Figure 2I and Figure S2C).

### 3.2. Machine Learning and MR-Based Identification of Key Druggable Genes

**Figure 3:**
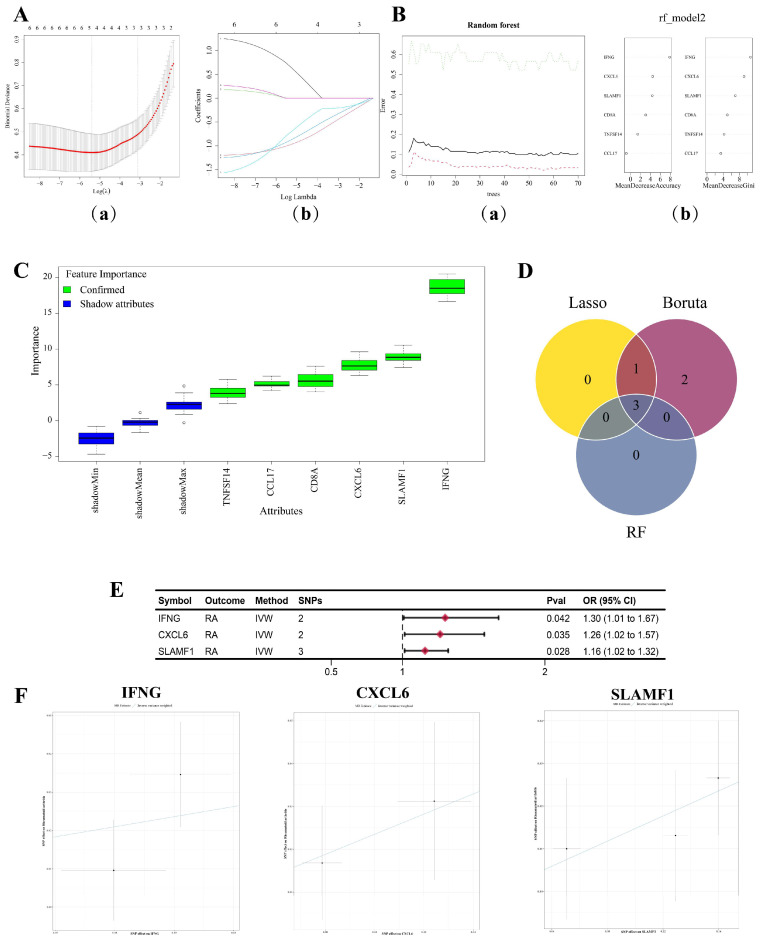
Machine Learning-Driven Identification and Mendelian Randomization Analysis of Three Key Druggable Genes. (**A**) (**a**,**b**) Key druggable genes identified by LASSO, based on binomial deviance, cross-validation, and gene coefficient paths; in (**a**), the red curve and gray error bars represent the mean cross-validated binomial deviance and ±1 standard error, respectively, while the two vertical dashed lines indicate the minimum-error and one-standard-error criteria; in (**b**), each colored line represents the coefficient path of an individual gene, and genes with nonzero coefficients at the penalty parameter selected by the minimum-error criterion were retained. (**B**) (**a**,**b**) RF model performance and feature importance, with error curves and ranking of candidate genes by MeanDecreaseAccuracy and MeanDecreaseGini; in (**a**), the black solid line represents the overall out-of-bag (OOB) error rate, while the red dashed and green dotted lines represent the class-specific OOB error rates for the RA and control groups, respectively; in (**b**), the open circles indicate the MeanDecreaseAccuracy and MeanDecreaseGini scores of individual genes, and the dotted horizontal lines serve as visual guides. (**C**) Feature selection using the Boruta algorithm, showing the importance distribution of candidate and shadow features; the green boxplots represent confirmed important genes, while the blue boxplots represent shadow attributes used as reference variables, and no tentative or rejected features were identified. (**D**) Venn diagram of the three key druggable genes consistently identified by LASSO, RF, and Boruta algorithms. (**E**) Forest plot showing the causal effects of the three key targets on RA. (**F**) Scatter plot illustrating the associations between *IFNG, CXCL6, SLAMF1*, and RA risk; the black points represent individual instrumental SNPs, the horizontal and vertical error bars indicate the corresponding standard errors, and the blue lines represent the IVW regression estimates, with positive slopes indicating positive causal associations with RA risk.

Based on the training set, the LASSO penalized regression model was applied to the six candidate druggable genes. The optimal λ value was selected through ten-fold cross-validation based on binomial deviance (Figure 3A). At the chosen optimal λ, those genes (*CD8A*, *IFNG*, *CXCL6*, and *SLAMF1*) showing nonzero parameter estimates were identified as potential key genes. RF analysis showed stable prediction error and highlighted *IFNG*, *CXCL6*, and *SLAMF1* as the most important features based on Mean Decrease Accuracy and Gini metrics (Figure 3B). The Boruta algorithm further confirmed all six genes as relevant predictors, with the confirmed features exhibiting markedly higher importance than random references (Figure 3C). Collectively, cross-validation across LASSO, RF, and Boruta consistently identified *IFNG*, *CXCL6*, and *SLAMF1* as robust key druggable genes for subsequent analysis (Figure 3D).

Two-sample MR analysis was conducted to confirm whether these genes had a causal impact on susceptibility to RA. Using the IVW method, *IFNG* (OR = 1.30, 95% CI: 1.01–1.67, *p* = 0.042), *CXCL6* (OR = 1.26, 95% CI: 1.02–1.57, *p* = 0.035), and *SLAMF1* (OR = 1.16, 95% CI: 1.02–1.32, *p* = 0.028) were all significantly associated with increased RA susceptibility (Figure 3E,F).

### 3.3. Expression Validation, Functional Characterization, and Clinical Prediction of Three Druggable Genes

**Figure 4:**
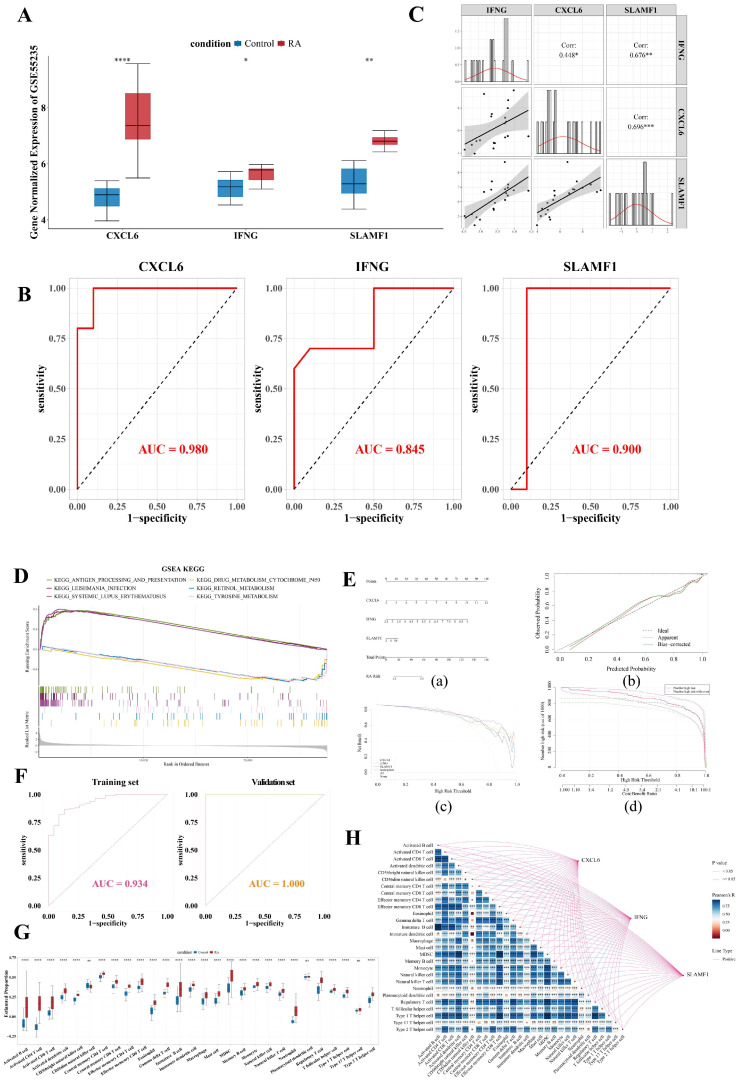
Expression Patterns, Diagnostic Evaluation, and Functional Immune Mechanisms of Three Druggable Genes in RA. (**A**) Box plots showing differential expression of *IFNG*, *CXCL6*, and *SLAMF1* between RA patients and controls; ns, not significant; * *p* < 0.05, ** *p* < 0.01, *** *p* < 0.001, and **** *p* < 0.0001. (**B**) ROC curves illustrating the diagnostic performance of the three genes in the validation set. (**C**) Correlation analysis of the expression levels among the three genes in the validation set. (**D**) Single-gene GSEA based on *CXCL6* expression groups. (**E**) (**a**–**d**) Nomogram, calibration curve, DCA curve, and CIC curve for the three druggable genes; in (**d**), the pink solid and green dashed curves represent the numbers of individuals classified as high risk and high-risk individuals with the event, respectively, while the corresponding outer curves indicate the confidence intervals. (**F**) ROC curve analysis of the nomogram model. (**G**) Box plot of immune cell infiltration in RA and control groups based on ssGSEA. (**H**) Correlation between the expression of the three druggable genes and immune cell infiltration.

To validate the biological and clinical relevance of *IFNG*, *CXCL6*, and *SLAMF1*, we conducted differential expression and ROC curve analysis using the training (GSE89408) and validation (GSE55235) datasets. All three genes were significantly overexpressed in RA samples compared with HC, with consistent expression trends across both datasets (Figure S3A and Figure 4A). ROC curves demonstrated excellent diagnostic performance, with AUC values > 0.8 in both datasets (training: *IFNG* = 0.879, *CXCL6* = 0.891, *SLAMF1* = 0.874; validation: *IFNG* = 0.845, *CXCL6* = 0.980, *SLAMF1* = 0.900; Figure S3B and Figure 4B). Correlation analysis showed a strong positive correlation among the three genes, especially between *SLAMF1* and *IFNG*. The correlation coefficient r was 0.880 in the training set and 0.676 in the validation set (Figure S3C and Figure 4C). This suggests that they may exert potential synergistic effects in the pathogenesis of RA.

We used GSEA to assess pathway enrichment. The high-expression groups defined for the three genes were significantly enriched in immune and metabolism-related pathways, including antigen processing and presentation, cytokine–cytokine receptor interaction, and drug metabolism by cytochrome P450 (Figure 4D and Figure S4A,B). The enriched pathways are important modulators of immune activation, cytokine signaling, and metabolic pathways. This finding indicates that these druggable genes (*IFNG, CXCL6, SLAMF1*) may contribute to RA progression by modulating immune responses and inflammatory signaling.

We constructed a nomogram model with the expression of the three marker genes to assess its predictive ability. The Bootstrap-based verification indicated good calibration and robustness of the nomogram model. DCA and CIC evaluations demonstrated good clinical benefit and risk stratification (Figure 4E). ROC analysis showed excellent separability, with AUCs of 0.934 and 1.000 for the training and validation cohorts, respectively (Figure 4F). This shows that the model has high predictive accuracy and clinical applicability.

Moreover, ssGSEA profiling identified marked differences in immune cell infiltration between RA and controls (25 different immune cell types, such as activated B cells and CD4+/CD8+T cells, were found to be differentially abundant) (Figure 4G). The expression of *IFNG*, *CXCL6*, and *SLAMF1* was positively associated with several immune cell subsets, including Th1 and Th2 cells, suggesting that higher expression of these genes may be associated with greater immune activation in RA (Figure 4H).

### 3.4. Single-Cell Landscape of RA T Cells Reveals Effector T Cell Dominance and Functional Correlation with Three Druggable Genes

**Figure 5:**
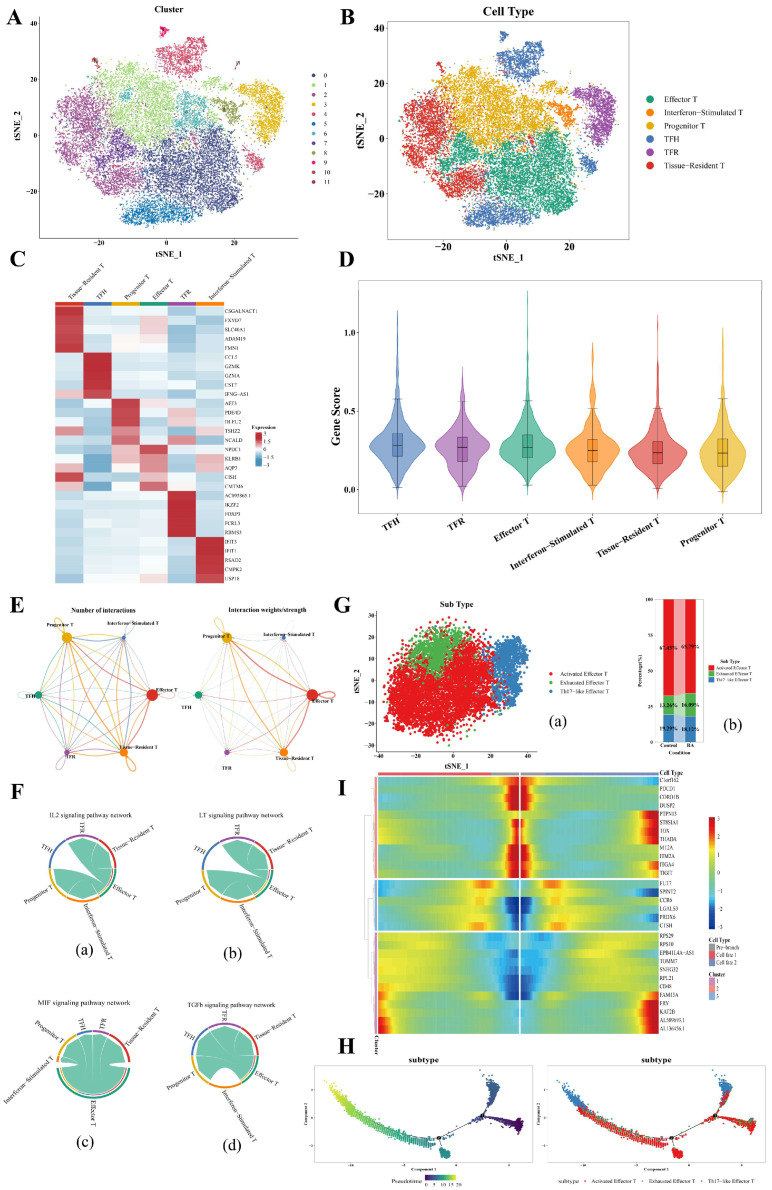
Single-Cell Data Annotation and Functional State Assessment of Effector T Cells. (**A**) t-SNE plot of cell clustering analysis. (**B**) Identification of six annotated cell types from 12 cell clusters. (**C**) Heatmap of marker genes for six cell types identified by FindAllMarkers analysis. (**D**) Visualization of differential scores of three druggable genes across six cell types in RA patients and controls. (**E**) Visualization of cell–cell communication connectivity, including the number and intensity of interactions among immune cell populations. (**F**) (**a**–**d**) Interactions between Effector T cells and other cell types mediated by key signaling pathways: IL-2, LT, MIF, and TGF-*β*. (**G**) (**a**,**b**) Distribution and proportions of Effector T-cell subtypes; in (**a**), the t-SNE plot shows the distribution of Activated Effector T, Exhausted Effector T, and Th17-like Effector T cells; in (**b**), the stacked bar plot shows the proportions of these subtypes in the control and RA groups. (**H**) Pseudotime differentiation trajectory of Effector T cell subpopulations; cells are colored by pseudotime in the left panel and by subtype in the right panel, and the numbered black circles indicate branch points in the inferred trajectory. (**I**) Heatmap illustrating the expression of top marker genes along differentiation trajectories of Effector T cell subtypes based on BEAM analysis.

Single-cell transcriptomic analysis was conducted on 6 samples (3 RA, 3 controls) to delineate T cell heterogeneity and explore the association between cell states and druggable gene expression. After quality control, normalization, and PCA-based dimensionality reduction (Figure S5A–C), 12 clusters were identified and annotated into six T cell subtypes: effector, interferon-stimulated, progenitor, Tfh, follicular regulatory T (Tfr), and tissue-resident T cells (Figure 5A,B). Effector T cells represented the predominant subtype in both groups and were markedly expanded in RA, whereas tissue-resident T cells were reduced (Figure S6A).

FindAllMarkers analysis highlighted diverse differentiation states and functional characteristics (Figure 5C). When comparing RA with HC, the scoring results differed significantly (*p* = 1.6551 × 10^−13^, Figure S6B), with Effector T cells exhibiting the highest scores among all cell types in RA patients (Figure S6C). The three druggable genes (*IFNG*, *CXCL6*, and *SLAMF1*) also showed peak scores in Effector T cells (Figure 5D), suggesting that these cells have a pivotal function in driving RA development, while also serving as valuable candidates for further mechanistic and pathway analyses. Expression profiling across six cell types revealed that *CXCL6* is weakly expressed, *IFNG* is predominantly expressed in Tfh and Progenitor T cells, and *SLAMF1* is uniformly expressed in all cell types (Figure S6D).

We used CellChat to analyse intercellular communication. Results indicated that the strongest signal connectivity was around effector T cells, which were also the central hub of cells with the strongest connectivity, with progenitor and tissue-resident cells contributing to this connectivity (Figure 5E and Figure S7A). Signaling pathways underlying this crosstalk included the transforming growth factor beta (TGF-β), macrophage migration inhibitory factor (MIF), lymphotoxin (LT), and interleukin-2 (IL-2) axes (Figure 5F and Figure S7B). Effector T cells are the main influencers and mediators in the RA immune environment through these pathways of immune activation, fibrosis and inflammatory feedback.

Using PCA clustering, we studied effector T cell subpopulations and identified marker genes specific to each subpopulation. These cells were then categorized into three functional subpopulations: Activated, Exhausted, and Th17-like (Figure 5G). *C1orf162* and *RPS10* were the top 2 marker genes for Activated, *PDCD1* and *TOX* were the top 2 marker genes for Exhausted, and *LGALS3* and *CTSH* were the top 2 marker genes for Th17-like cells, as shown in a heatmap (Figure S7C). Comparing the characteristic scores of druggable genes between the RA and control groups, box plots showed significant differences in the Activated and Th17-like groups, but not in the Exhausted group (Figure S7D). From these observations, changes in druggable gene expression are mostly associated with Activated and Th17-like Effector T cell states.

Using the Monocle algorithm for pseudotime trajectory analysis, we found that within T cells, there was dynamic evolution with the progression of RA. Initially, these cells had an activated phenotype and subsequently evolved into Th17-like and exhausted phenotypes (Figure 5H). Furthermore, we identified important genes (e.g., *C1orf162, PDCD1, CORO1B*) that differentiate between these trajectories using branched expression analysis modeling (BEAM) (Figure 5I). Three druggable genes were found to be stably expressed during effector T cell differentiation, suggesting they maintain constant functional roles (Figure S7E).

### 3.5. Potential TCM Screening and Active Ingredient Prediction

Through database mining with COREMINE, candidate TCM compounds targeting the three key druggable genes were identified. Subsequently, their pharmacokinetic and toxicity properties were evaluated using the TCMSP and ADMETlab platforms. The compound (+)-Ganoderic acid Mf demonstrated favorable safety along with physicochemical characteristics, pointing to notable therapeutic promise. Nevertheless, its substantial molecular mass may restrict systemic uptake (Figure S8).

### 3.6. Binding Stability Characteristics of Protein-Ligand Complexes

**Figure 6:**
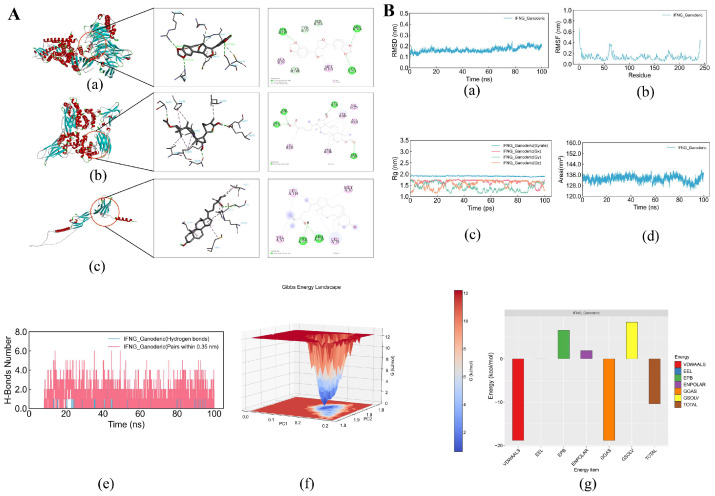
Screening of potential herbal medicines and prediction of active ingredients. (**A**) Molecular docking poses and interaction diagrams of three protein–compound complexes: (**a**) *CXCL6*–Sesamin, (**b**) *IFNG*–(+)-Ganoderic acid Mf, and (**c**) *SLAMF1*–(24R)-Saringosterol. Each panel shows the overall docking pose, enlarged binding site, and 2D interaction diagram; green, light green, and pink denote conventional hydrogen bonds, carbon/π-donor hydrogen bonds, and alkyl/π-alkyl interactions, respectively. (**B**) (**a**–**g**) The molecular dynamics simulation of the *IFNG* and (+)−Ganoderic acid Mf complex.

Molecular docking revealed strong binding affinities between *CXCL6*-Sesamin (−10.2 kcal/mol), *IFNG*-(+)-Ganoderic acid Mf (−8.3 kcal/mol), and *SLAMF1*-(24R)-Saringosterol (−6.4 kcal/mol) (Figure 6A).

In addition, molecular dynamics simulations supported the stability of the *IFNG*-(+)-Ganoderic acid Mf complex (Figure 6B). The RMSD values were within the range of 0.1 nm to 0.3 nm, and the RMSF values of the residues were below 0.4 nm, indicating good structural stability. The radius of gyration (1–2 nm) and SASA (120–140 nm^2^) remained relatively stable, thus supporting the complex’s compact structure. The number of hydrogen bonds showed slight fluctuations but remained generally stable. The free energy landscape revealed deep minima, and the molecular mechanics Poisson–Boltzmann surface area calculation yielded a binding free energy of −9.8 kcal/mol, indicating strong and stable binding between the protein and ligand. Overall, the results suggest that (+)-Ganoderic acid Mf and *IFNG* may form a stable complex with a favorable predicted binding energy, supporting further evaluation of (+)-Ganoderic acid Mf as a candidate compound for RA.

## 4. Discussion

GWAS and related studies based on QTLs have identified many genes associated with RA risk. However, these studies lack causal inference and integrated functional characterization. A major strength of this study is its multidimensional analytical framework. We integrated eQTL/pQTL-based MR, bulk transcriptomic analysis, machine-learning approaches, and batch-corrected single-cell transcriptomic analysis to identify and characterize candidate therapeutic targets for RA, and further screened TCM compounds as an exploratory extension. Although QTL data derived from peripheral blood are not necessarily representative of synovial tissue inflammation or intracellular protein function, this limitation was partially mitigated by cell type-resolved analyses, intercellular communication mapping, and multi-level validation. This framework strengthens causal evidence, provides a better understanding of the underlying mechanisms, and connects target discovery with potential clinical translation, thereby providing a robust and flexible approach to target identification in complex autoimmune diseases.

*CXCL6* (granulocyte chemotactic protein 2 [GCP-2]) is an important factor in immune activation and can recruit neutrophils, participating in innate immunity [34]. The upregulation of *CXCL6* is also important in RA, as it contributes to the local inflammatory response and disease progression, driven by synovitis, cartilage destruction, and large numbers of neutrophils in the synovial fluid [35,36]. *IFNG*, encoded on chromosome 12, is a key cytokine in immune regulation [37]. In the RA synovium, it is strongly expressed by clonally expanded T peripheral helper (Tph) cells, mediating Tph–B cell interactions via the *IFNG*-interferon gamma receptor (IFNGR) axis, thereby promoting B cell activation and contributing to tissue damage [38]. Excessive *IFNG* activation is recognized as a central driver of autoimmune pathogenesis and a promising therapeutic target [39]. *SLAMF1*, widely expressed on immune cells, shows elevated expression in the peripheral blood of patients with early RA [40]. In the collagen-induced arthritis mouse model, it is markedly upregulated in multiple immune cell types, including cytotoxic T lymphocytes, T helper cells, natural killer cells, natural killer T cells, conventional dendritic cells, and monocytes/macrophages [41].

In this study, *IFNG*, *CXCL6*, and *SLAMF1* were significantly positively correlated in RA (e.g., the correlation coefficient between *SLAMF1* and *IFNG* reached 0.880), suggesting coordinated regulation. Mechanistically, lipopolysaccharide and tumor necrosis factor alpha (TNF-α) promote the secretion of *CXCL6* [42], which may exacerbate inflammation by recruiting neutrophils. IFNG may enhance autoantibody production via Tph-B cell interactions [43]. Elevated Tfh and Tph cells in RA, modulated by treatment, may serve as predictive biomarkers for biotherapy response [44]. *SLAMF1* may potentiate both effects by promoting T cell activation and intercellular communication. In anti-tuberculosis immunity, *SLAMF1* promotes macrophage-T cell interactions and enhances reactive oxygen species generation, thereby exerting protective effects against tuberculosis [45]. Single-cell RNA sequencing further revealed preferential *IFNG* expression in follicular helper and naïve T cells, alongside broad *SLAMF1* distribution across diverse T cell subsets. The TGF-β signaling pathway is highly expressed in progenitor T cells, which may be associated with its role in early inhibition of autoimmune responses, promotion of Treg cell differentiation, and maintenance of immune homeostasis. By reducing interleukin-6 (IL-6) R expression, TGF-β inhibits the IL-6 signaling pathway, thereby suppressing inflammatory responses, consistent with previously reported findings [46]. It also clarified the differentiation trajectories of effector T cells and their intercellular communication via pathways like MIF and IL-2, amplifying local inflammatory signaling. Innate immune activation and adaptive immune dysregulation are connected through this integrated network of chemokines, cytokines and immune costimulatory molecules. It offers a multi-target, integrated perspective on the amplification of the inflammatory cascade in RA to support the identification of new therapeutic targets.

These results might be used to group patients by biomarker profile in routine clinical practice. High diagnostic and predictive performance was achieved with ROC and nomogram analyses (AUC = 0.934 in the training set and 1.000 in the validation set). Differentiated patient subgroups can be targeted for therapeutic strategies. In synovitis that has a neutrophilic predominance, for example, antagonizing *CXCL6* could be beneficial, while reducing *IFNG*/IFNGR signaling could help to dampen T-cell–B-cell crosstalk–driven autoimmunity. These therapies would be in addition to existing anti-TNF-α or IL-6 therapies. scRNA-seq was used to obtain high-resolution cell-type classification, revealing that IFNG was specifically enriched in Tfh cells. At the same time, *SLAMF1* was broadly expressed across all T cell types. These insights, in turn, help more finely define the specific cell populations that such treatment strategies would target.

As for translational research, molecular docking suggested that compounds including sesamin, Ganoderic acid Mf, and (24R)-saringosterol derived from TCM were predicted to interact with *CXCL6*, *IFNG*, and *SLAMF1*, respectively. The stable binding interaction between *IFNG* and Ganoderic acid Mf, as revealed by MD simulations, suggests the therapeutic potential of this complex. As RA is a disease with multiple etiological factors, the above-mentioned compounds with multiple molecular targets are well suited and warrant further preclinical validation. There are existing studies on Sesamin and Ganoderic Acid [47,48]. A combination of network pharmacology and molecular docking is a feasible approach. With this approach, the therapeutic merits of TCM can be integrated into modern precision immunotherapies. Cellular and animal-level experiments will be conducted to evaluate the therapeutic potential of these compounds.

Despite the strengths of the integrative multi-omics design, several limitations of our study should be acknowledged. First, our reliance on publicly available datasets inevitably introduces heterogeneity in sample characteristics and experimental platforms, which may constrain the broader applicability of our findings. Second, although informative, the single-cell analysis was based on a small cohort comprising only three patients with RA and three HC. Moreover, the single-cell dataset was derived from peripheral blood Tfh cells and may not fully recapitulate the synovial microenvironment. Accordingly, the single-cell findings should be regarded as hypothesis-generating rather than conclusive. Third, although the identified genes were validated in an independent transcriptomic dataset, the relatively small sample size calls for further assessment in larger and more diverse cohorts. Finally, although multiple computational analyses support the biological relevance of these targets and the predicted target–compound interactions, experimental validation in cellular and animal models is warranted before definitive conclusions can be drawn.

## 5. Conclusions

In summary, this study combined multiple layers of information to identify three druggable genes, *CXCL6*, *IFNG*, and *SLAMF1*, with potential causal associations with RA and favorable diagnostic performance. Single-cell analyses further characterized their cell type-specific expression patterns and potential functional relevance to immune activation and intercellular communication. In silico analyses also prioritized sesamin, (+)-Ganoderic acid Mf, and (24R)-saringosterol as candidate compounds for further experimental evaluation. This integrative approach provides a basis for identifying novel therapeutic targets and supporting future mechanistic and translational studies in RA.

## Data Availability

The datasets used and/or analysed during the current study are available from the corresponding author on reasonable request.

## Supplementary Materials

The following supporting information can be downloaded at: https://www.mdpi.com/article/10.3390/genes17070828/s1, Figure S1: WGCNA analysis. (A) Dendrogram showing the hierarchical clustering of samples. (B) Plot illustrating the relationship between soft-thresholding power values (ranging from 1 to 30) and the scale-free topology fit index. The *x*-axis indicates the power parameter, while the *y*-axis (left) displays the scale-free topology model fit, represented by signed R^2^. Higher values of signed R^2^ indicate a closer approximation to a scale-free network. (C) Plot showing the effect of soft-thresholding power values (1–30) on the average adjacency among all genes within each gene module. The *x*-axis represents the power parameter, and the *y*-axis (right) indicates the mean adjacency; Figure S2: Functional enrichment analysis and PPI network. (A) Bubble plot of GO enrichment analysis for the five protective genes. (B) Bubble plot of KEGG pathway enrichment analysis for the five protective genes. (C) STRING-based PPI network of the 20 genes; Figure S3: Expression and ROC analysis of three druggable genes. (A) Box plot illustrating statistically significant differences in the expression levels of three druggable genes between RA and control groups. (B) ROC curves demonstrating the discriminatory ability of the three druggable genes in the training set. (C) Correlation plot depicting the relationships among the expression levels of the three druggable genes in the training set; Figure S4: GSEA analysis. (A) Single-gene GSEA illustrating pathway enrichment based on *IFNG* expression level groups. (B) Single-gene GSEA illustrating pathway enrichment according to *SLAMF1* expression level groups; Figure S5: Single-cell data analysis comparing RA and control groups. (A) (a,b) Comparison of cell distributions before and after quality control. (B) (a,b) PCA of highly variable genes, distinguishing samples by original source and condition. (C) PCA elbow plot indicating the optimal number of principal components for further analysis; Figure S6: t-SNE analysis and druggable gene scoring in RA and control samples. (A) (a,b) t-SNE analysis of six cell types in RA patients and controls, with proportional distributions shown in stacked bar plots. (B) Comparison of expression scores of three druggable genes between RA patients and controls in the entire dataset. (C) Comparison of expression scores of the three druggable genes between RA patients and controls within each of the six cell types. (D) Visualization of the expression patterns of the three druggable genes mapped onto the cell atlas; Figure S7: Analyses of effector T cell communication, markers, and druggable gene expression. (A) Ligand-based bubble plot illustrating communication between effector T cells and other cell types. (B) (a–d) Cell–cell communication analysis among six immune cell types across the IL-2, LT, MIF, and TGF-β signaling pathways. (C) Heatmap showing the top five marker genes for effector T cell subtypes identified by FindAllMarkers analysis. (D) Box plots comparing the expression scores of three druggable genes among Activated Effector T, Exhausted Effector T, and Th17-like Effector T subpopulations between RA and control groups. (E) Dynamic expression profiles of the three druggable genes along differentiation trajectories of effector T cell subtypes; Figure S8: Pharmacological evaluation of three druggable genes. Line plots showing the pharmacological properties and environmental toxicity scores of (+)-Ganoderic acid Mf, (24R)-Saringosterol, and Sesamin; Table S1: Datasets used in this study.

## Abbreviations

The following abbreviations are used in this manuscript:

AUC	Area Under the ROC Curve
ADMET	Absorption, Distribution, Metabolism, Excretion, and Toxicity
BEAM	Branched Expression Analysis Modeling
CIC	Clinical Impact Curves
DCA	Decision Curve Analysis
DEGs	Differentially Expressed Genes
DL	Drug-Likeness
eQTL	Expression Quantitative Trait Locus
GEO	Gene Expression Omnibus
GO	Gene Ontology
GSEA	Gene Set Enrichment Analysis
GWAS	Genome-Wide Association Study
HC	Healthy Controls
IEU	Integrative Epidemiology Unit
IL-2	Interleukin-2
IL-6	Interleukin-6
IL-17	Interleukin-17
IVW	Inverse-Variance Weighted
KEGG	Kyoto Encyclopedia of Genes and Genomes
LASSO	Least Absolute Shrinkage and Selection Operator
LD	Linkage Disequilibrium
LT	Lymphotoxin
MD	Molecular Dynamics
MIF	Macrophage Migration Inhibitory Factor
MR	Mendelian Randomization
NF-κB	Nuclear Factor Kappa B
OB	Oral Bioavailability
ORs	Odds Ratios
PCA	Principal Component Analysis
PDB	Protein Data Bank
PPI	Protein–Protein Interaction
pQTL	Protein Quantitative Trait Locus
RA	Rheumatoid Arthritis
RF	Random Forest
Rg	Radius of Gyration
RMSD	Root Mean Square Deviation
RMSF	Root Mean Square Fluctuation
ROC	Receiver Operating Characteristic
SASA	Solvent-Accessible Surface Area
scRNA-seq	Single-Cell RNA Sequencing
ssGSEA	Single-Sample Gene Set Enrichment Analysis
TCM	Traditional Chinese Medicine
TCMSP	Traditional Chinese Medicine Systems Pharmacology Database
Tfh	Follicular Helper T Cells
Tfr	Follicular Regulatory T
TGF-β	Transforming Growth Factor Beta
TNF-α	Tumor Necrosis Factor Alpha
TOM	Topological Overlap Matrix
Tph	T Peripheral Helper Cells
WGCNA	Weighted Gene Co-Expression Network Analysis
