# Supplementary material for: Identification of Potential Biomarkers for Rheumatoid Arthritis Based on Integrated Bioinformatics and Single-Cell RNA-Seq
Source: Genes (Basel). 2026 Jul 21;17(7):828. doi: 10.3390/genes17070828 (PMC13409446; doi:10.3390/genes17070828)
Supplement: Supplementary file 1 [file genes-17-00828-s001.zip › genes-4428148-supplementary.pdf]

SUPPLEMENTARY MATERIAL

Identification of potential biomarkers for rheumatoid arthritis based on integrated bioinformatics and single-cell RNA-seq

Jinling Zhang[1,3], Ke Han*[1,2,3]

Jinling Zhang [1,2] and Ke Han [1,2,3,*]

1 School of Pharmacy, Harbin University of Commerce, Harbin 150076, China; zhangjinling194029@126.com
2 Heilongjiang Provincial Key Laboratory of Fluid Engineering Equipment and Digital Intelligence
Technology, Harbin University of Commerce, Harbin 150029, China
3 School of Computer and Information Engineering, Harbin University of Commerce, Harbin 150028, China
* Correspondence: hanke@hrbcu.edu.cn

Figures

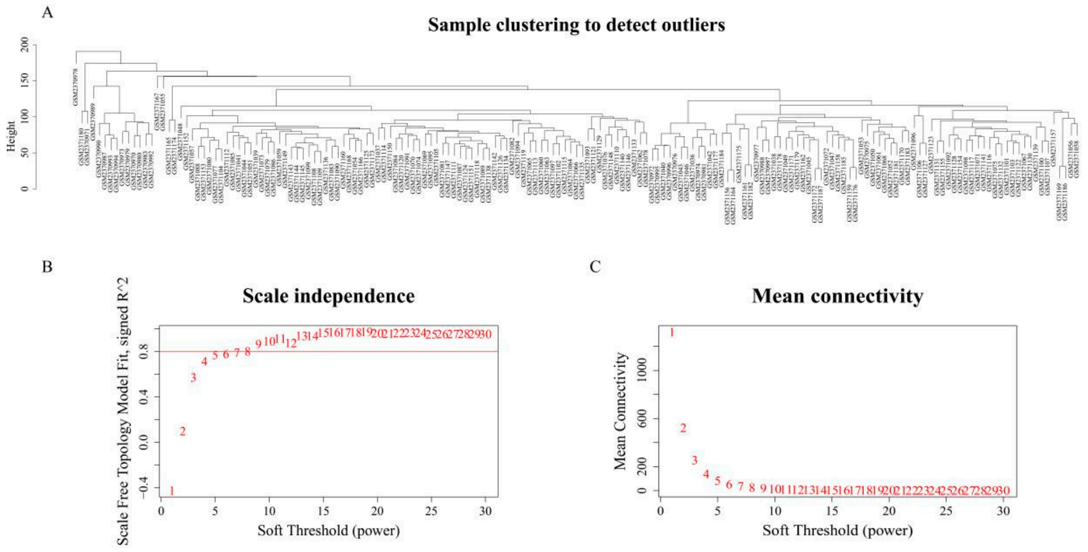

Figure S1. WGCNA analysis. **(A)** Dendrogram showing the hierarchical clustering of samples. **(B)** Plot illustrating the relationship between soft-thresholding power values (ranging from 1 to 30) and the scale-free topology fit index. The x-axis indicates the power parameter, while the y-axis (left) displays the scale-free topology model fit, represented by signed R^2. Higher values of signed R^2 indicate a closer approximation to a scale-free network. **(C)** Plot showing the effect of soft-thresholding power values (1-30) on the average adjacency among all genes within each gene module. The x-axis represents the power parameter, and the y-axis (right) indicates the mean adjacency.

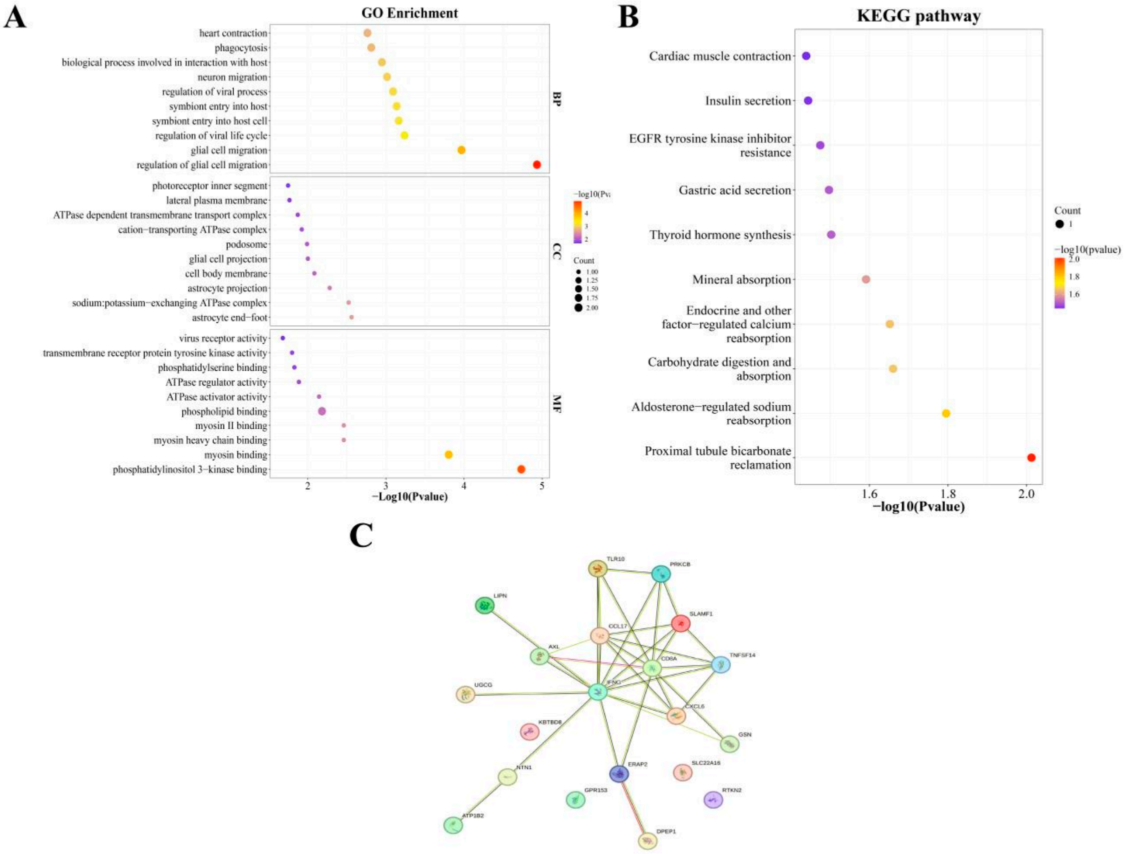

Figure S2. Functional enrichment analysis and PPI network. **(A)** Bubble plot of GO enrichment analysis for the five protective genes. **(B)** Bubble plot of KEGG pathway enrichment analysis for the five protective genes. **(C)** STRING-based PPI network of the 20 genes.

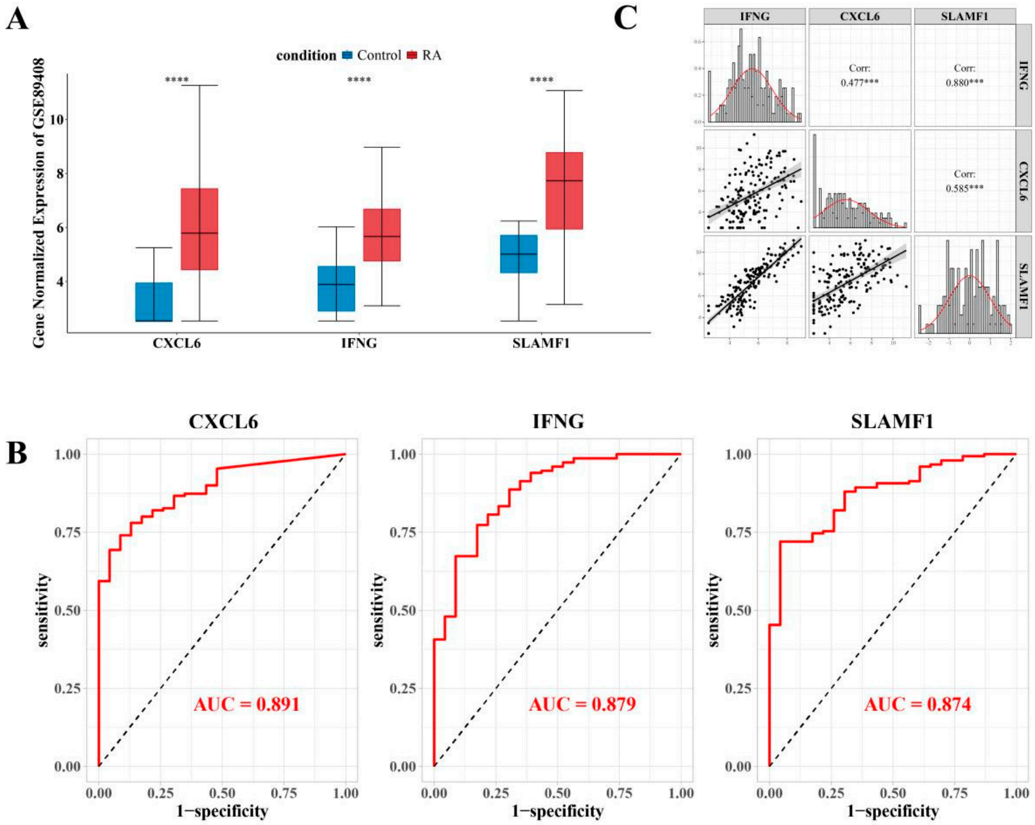

Figure S3. Expression and ROC analysis of three druggable genes. **(A)** Box plot illustrating statistically significant differences in the expression levels of three druggable genes between RA and control groups. **(B)** ROC curves demonstrating the discriminatory ability of the three druggable genes in the training set. **(C)** Correlation plot depicting the relationships among the expression levels of the three druggable genes in the training set.

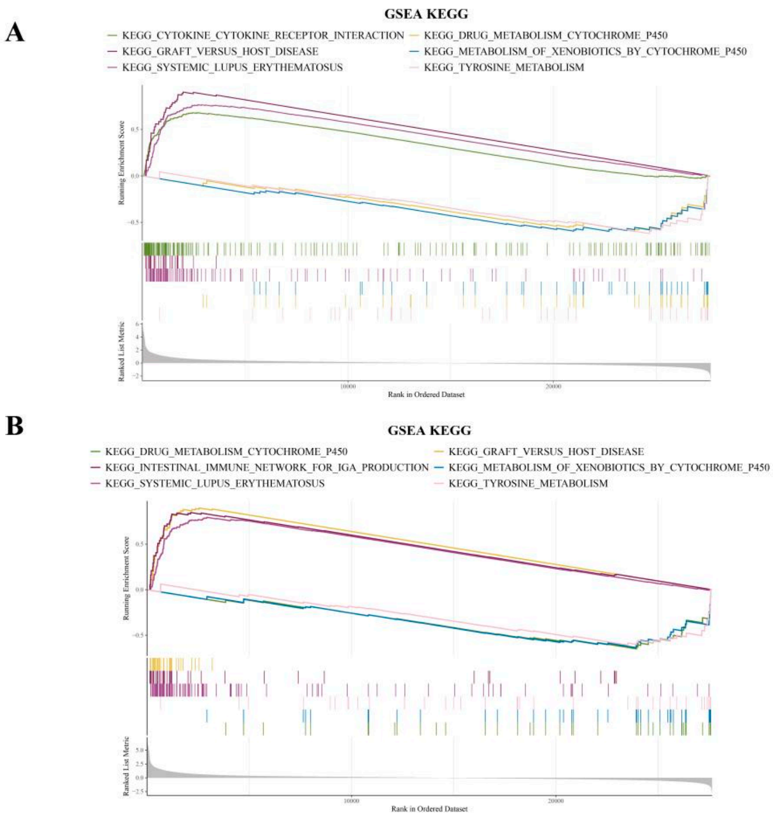

Figure S4. GSEA analysis. **(A)** Single-gene GSEA illustrating pathway enrichment based on IFNG expression level groups. **(B)** Single-gene GSEA illustrating pathway enrichment according to SLAMF1 expression level groups.

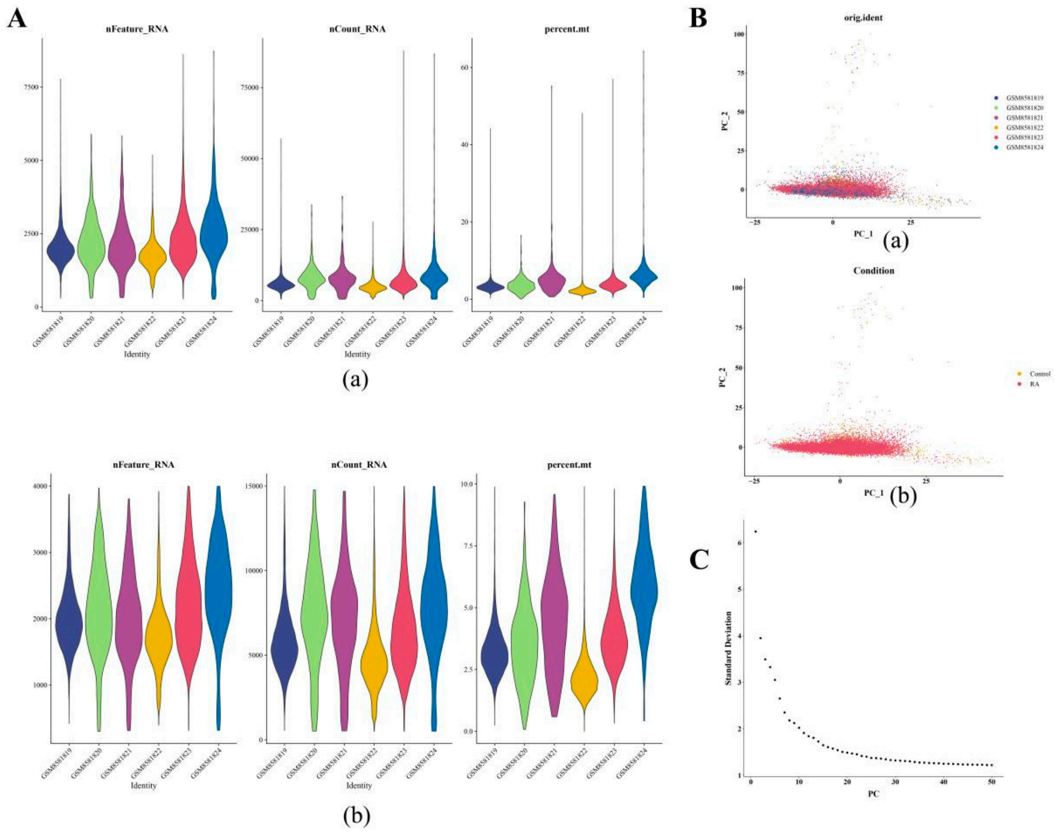

Figure S5. Single-cell data analysis comparing RA and control groups. **(A)** (a)-(b) Comparison of cell distributions before and after quality control. **(B)** (a)-(b) PCA of highly variable genes, distinguishing samples by original source and condition. **(C)** PCA elbow plot indicating the optimal number of principal components for further analysis.

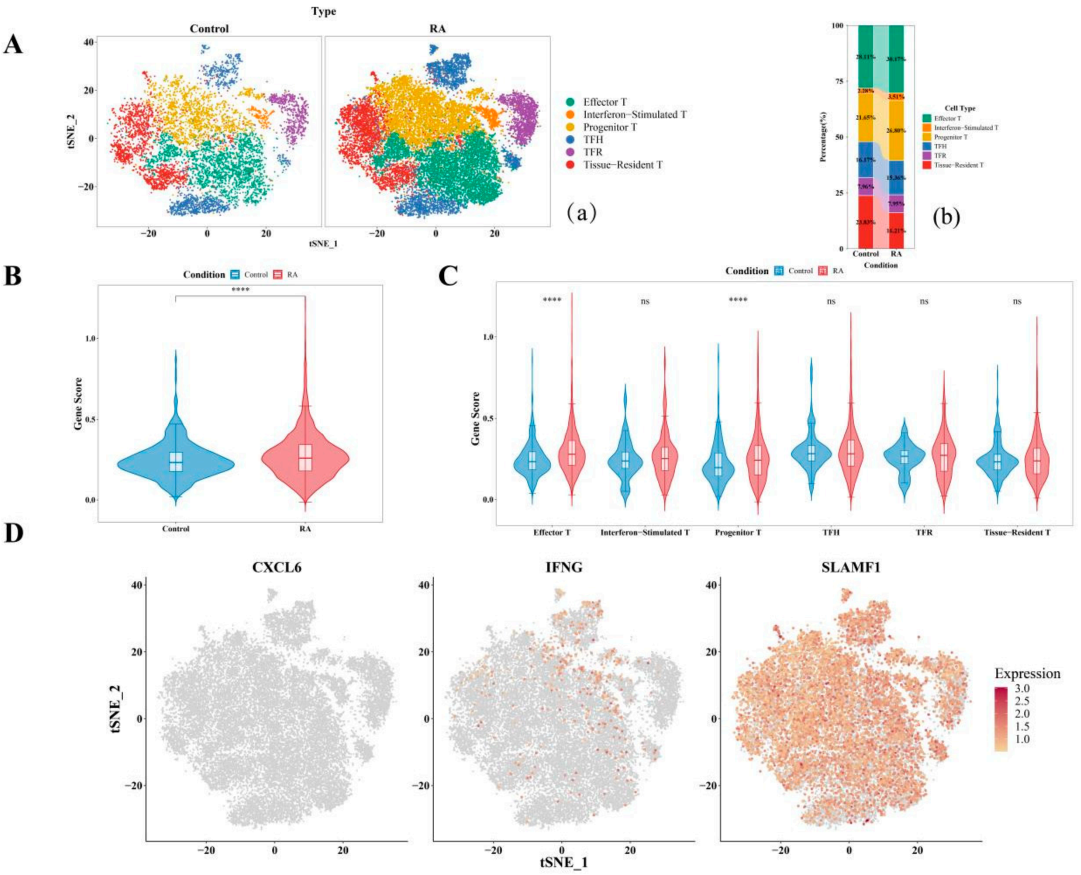

Figure S6. t-SNE analysis and druggable gene scoring in RA and control samples. **(A)** (a)-(b) t-SNE analysis of six cell types in RA patients and controls, with proportional distributions shown in stacked bar plots. **(B)** Comparison of expression scores of three druggable genes between RA patients and controls in the entire dataset. **(C)** Comparison of expression scores of the three druggable genes between RA patients and controls within each of the six cell types. **(D)** Visualization of the expression patterns of the three druggable genes mapped onto the cell atlas.

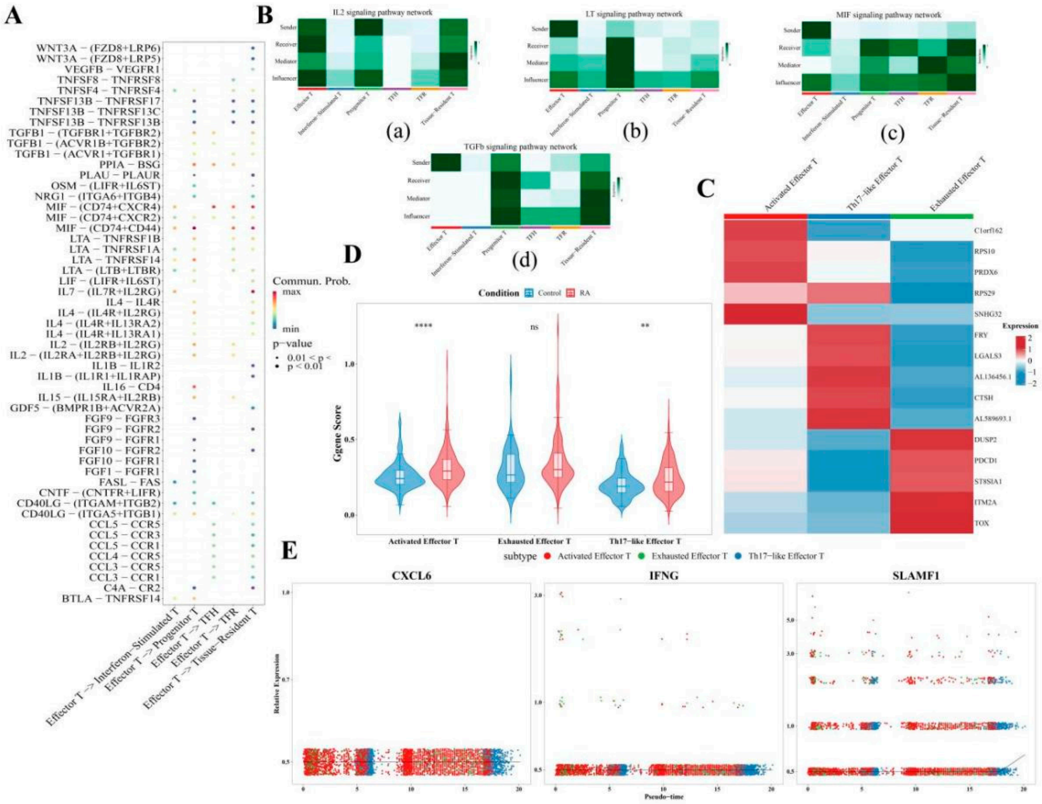

Figure S7. Analyses of effector T cell communication, markers, and druggable gene expression. **(A)** Ligand-based bubble plot illustrating communication between effector T cells and other cell types. **(B)** (a)-(d) Cell-cell communication analysis among six immune cell types across the IL2, LT, MIF, and TGF-b signaling pathways. **(C)** Heatmap showing the top five marker genes for effector T cell subtypes identified by FindAllMarkers analysis. **(D)** Box plots comparing the expression scores of three druggable genes among Activated Effector T, Exhausted Effector T, and Th17-like Effector T subpopulations between RA and control groups. **(E)** Dynamic expression profiles of the three druggable genes along differentiation trajectories of effector T cell subtypes.

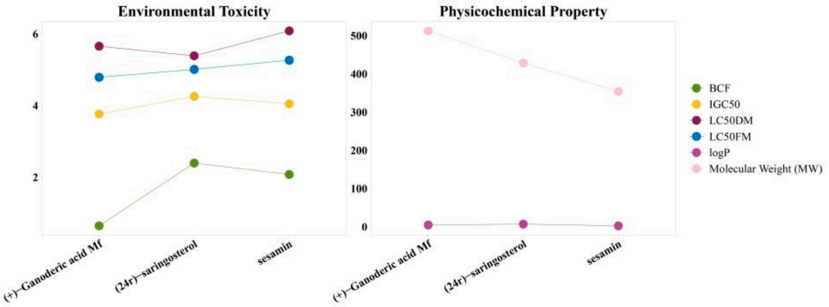

Figure S8. Pharmacological evaluation of three druggable genes. Line plots showing the pharmacological properties and environmental toxicity scores of (+)-Ganoderic acid Mf, (24R)-Saringosterol, and Sesamin.

Table

Table S1: Datasets used in this study

Accession ID	RA	Control	Group	Organism	Platform
GSE89408	150	23	Train	Homo sapiens	Illumina HiSeq 2000
GSE55235	10	10	Test	Homo sapiens	Ffymetrix Human Genome U133A Array
GSE279838	3	3	Single-cell	Homo sapiens	Illumina NovaSeq 6000
